# Single Medial Prefrontal Neurons Cope with Error

**DOI:** 10.1371/journal.pone.0006240

**Published:** 2009-07-17

**Authors:** Thomas Michelet, Bernard Bioulac, Dominique Guehl, Michel Goillandeau, Pierre Burbaud

**Affiliations:** CNRS UMR 5227 MAC Mouvement - Adaptation – Cognition, Université Victor Segalen Bordeaux 2, Bordeaux, France; L'université Pierre et Marie Curie, France

## Abstract

Learning from mistakes is a key feature of human behavior. However, the mechanisms underlying short-term adaptation to erroneous action are still poorly understood. One possibility relies on the modulation of attentional systems after an error. To explore this possibility, we have designed a Stroop-like visuo-motor task in monkeys that favors incorrect action. Using this task, we previously found that single neurons recorded from the anterior cingulate cortex (ACC) were closely tuned to behavioral performance and, more particularly, that the activity of most neurons was biased towards the evaluation of erroneous action. Here we describe single neurons engaged in both error detection and response alertness processing, whose activation is closely associated with the improvement of subsequent behavioral performance. Specifically, we show that the effect of a warning stimulus on neuronal firing is enhanced after an erroneous response rather than a successful one and that this outcome is correlated with an error rate decrease. Our results suggest that the anterior cingulate cortex, which exhibits this activity, serves as a powerful computational locus for rapid behavioral adaptation.

## Introduction

Learning often occurs by trial and error. After a success, the correct action is rapidly reinforced. Failure, on the other hand, leads the individual to make new attempts and to change strategies in order to reach the goal. Learning theories have focused mainly on the long-term synaptic potentiation of correct responses through positive reinforcement, but little is known about the neuronal mechanisms involved in error correction or behavioral compensation.

Such adaptive processes require at least evaluative and strategic functions involving error detection as well as behavior-correction mechanisms. Significant evidence suggests that the anterior cingulate cortex (ACC) plays a role in these aspects of behavioral learning. First, several studies indicate that this cortical area encodes performance evaluation. Since the observation of negative event-related potentials during incorrect behavioral responses [1], [2], a number of neuroimaging and electrophysiological studies in humans have suggested that the anterior cingulate cortex (ACC) is involved in error detection [3], [4]. This finding was confirmed at the neuronal level [5], [6], [7], and the role of evaluation-related neurons capable of responding to both positive and negative feedback was discussed in a recent paper [8]. Second, observations of persistent error in human patients after ACC lesion suggests that the ACC also plays a role in attentiveness processing [3], [4], [9], but only sparse evidence exists for a direct role for the ACC in performance adjustment. Moreover, these contributions are often presented in a mutually exclusive fashion, and the direct evidence for a link between these two functions is scant.

We have hypothesized that these two functions are closely interrelated, such that an attentional process could influence the outcome of a given action and/or the outcome could in turn act reciprocally on the subsequent attentional allocation.

**Figure 1:**
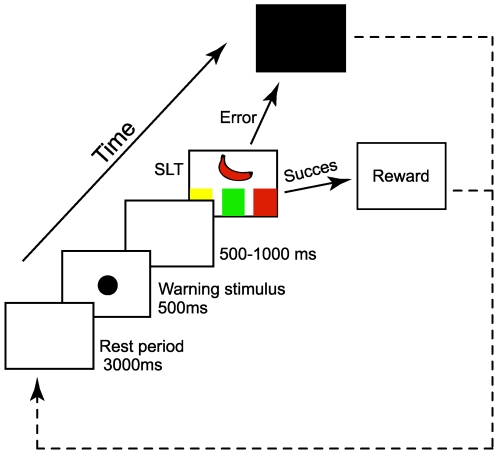
Stroop-like task (SLT) in monkey. After a 3000 ms rest period, a warning stimulus (black circular cue) was presented for 500 ms in the center of a video screen. The SLT target then appeared, followed by a drop of juice (successful response) or a black screen (erroneous response). Each stimulus interval was randomized between 500 and 1000 ms.

We designed a non-verbal monkey version of the original Stroop task [10], [11] that is known to favor incorrect responses and hence trigger behavioral adaptation (Figure 1; for details, see Materials and Methods and [8]). After a rest period, each trial began with the appearance of a black circular cue as a warning stimulus. Such stimuli are known to increase response readiness in preparation for an impending specific task [12]. At the end of each trial, a drop of juice was given if the monkey responded successfully, while a negative visual signal was provided after incorrect responses in order to indicate response failure. During such attentional and evaluative periods, the unitary activity of neurons was recorded in the most rostral part (CMAr or area 24c) of the cingulate motor areas [13], [14]. To assess the possibility of a causal link at the cellular level, we analyzed single-neuronal activity patterns in the ACC both during an ongoing behavioral trial and as a function of previous trial performance in monkeys trained to execute this highly attention-demanding task.

## Results

### Warning-related neurons

**Figure 2:**
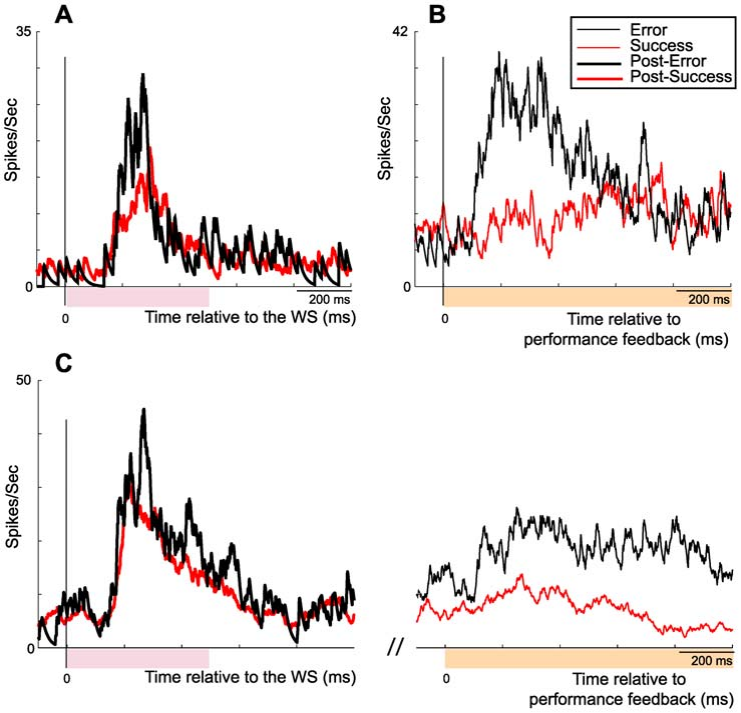
Neuronal responsive enhancement to salient stimuli. (A) Neuronal responsive enhancement to a warning stimulus (WS, pink color epoch) after an erroneous behavioral response: firing rate was enhanced during trials following an error response (thin black trace). (B) Evaluation-related (orange color epoch) ACC neuronal activity is biased towards error processing: neuronal activation is strong when time-locked to the error signal (thick black trace) but slight when locked to reward delivery (thick red trace) C) Firing rate changes of the same CMAr neuron during both the warning stimulus (left) and evaluative (right) period of repeated trials: post-error successful trials. Coloring as in C.

**Figure 3:**
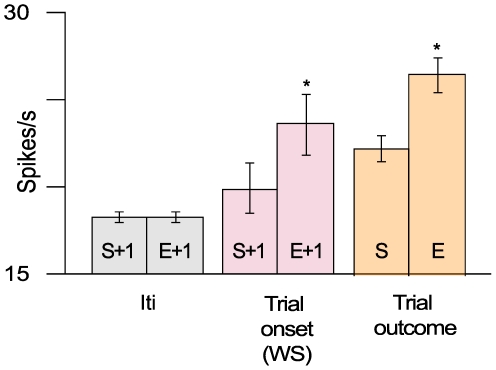
Mean firing rates in the recorded neuronal population. Right bars: increased firing rate for negative feedback (E) in comparison with positive feedback (S). Middle bars: warning stimulus (WS) responsiveness is enhanced after an erroneous behavioral response (E+1); left bars show virtually identical mean firing rates for inter-trial activity (Iti) measured immediately prior to WS presentation.

Among the 372 recorded neurons, 156 (42%) showed selective responses to the WS with none responding to non-contextual visual stimuli. Of this neuronal ensemble, 79 cells met the statistical criteria for comparison between post-success and post-error trial activities. An example of a recorded neuron exhibiting a post-error modification pattern is given in Figure 2A. The magnitude of a given WS-related neuron's increase in activity was significantly greater (*t*-test, *p*<0. 0001) after a previous erroneous action (23.6±1.8) than after a successful one (19.8±1.4) (Figure 3; pink middle histogram pair). To verify that this enhanced activity was not simply due to a global increase in cingulate activity, we compared the mean firing rates during the 3 s rest period, which approximately corresponded to the interval between two successive trials. Since there was no change (*t*-test, *P*>0.05) in resting activity during the course of the recording sessions (Figure 3; grey left histogram pair), we concluded that the post-error activity enhancement was specific to the WS influence, thereby providing the substrate for a functional link between error detection and a subsequent attentional process.

### Bimodal neurons

**Figure 4:**
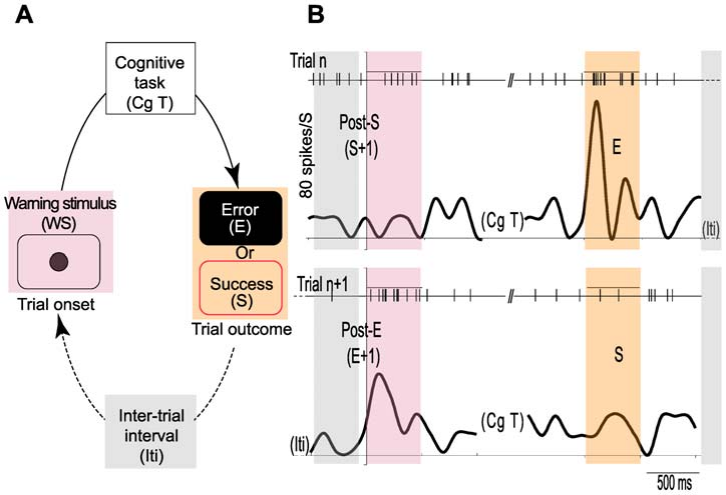
Bimodal neurons. (A) Schematic representation of the behavioral task. Trials always began with a warning stimulus (WS; black circle) and ended with an evaluative period (Trial outcome). Each trial followed the previous one after a 3 sec inter-trial interval (Iti). (B) Firing rate changes in a single ACC neuron during both the warning-stimulus (pink color) and the evaluative period (orange color) of two sequential trials. Each thin vertical bar represents an individual action potential and each curve represents the instantaneous firing rate of the neuron. The top panel shows post-success erroneous trials; the bottom panel, post-error successful trials. Coloring as in Figure 3.

In a previous paper, we described a CMAr neuronal population that responded during the evaluation period to both positive and negative feedback (see [8] and Figure 2B, orange right histogram pair). More than 20% of the recorded neurons (79 of 372) shared characteristics of evaluative-related and WS-related neurons by responding during both task phases (Figure 2C). Such “bimodal” neurons exhibited a small WS-related activation, indicating that the previous behavioral response had been correct, and subsequently that a larger phasic activation related to the negative feedback signal was indicative of a failed performance. Frequently, subsequent responses exhibited an increase in WS-related activity (indicative of post-error trials) and a smaller evaluative activity in response to reward delivery. As schematized in Figure 4, two successive trial responses of a recorded neuron exhibited both attentional- and evaluative-related activity according to a post-error effect. Such activity patterns demonstrate the link between evaluative and subsequent strategic functions at the cellular level within the CMAr.

### Behavioral adaptations

**Figure 5:**
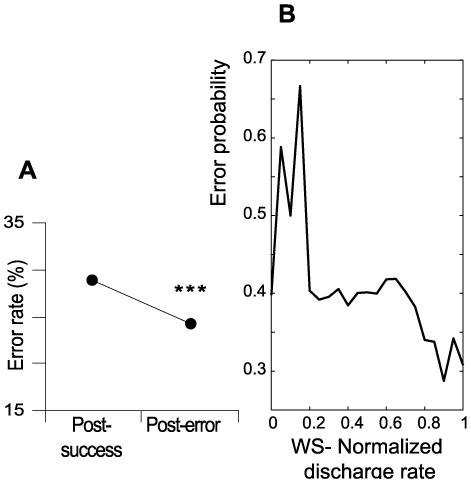
Adaptation to error. (A) Pooled data showing a lower behavioral error rate in post-error trials than in post-success trials (p<0.001). * *P*<0.01; ** *P*<0.001. (B) Based on trial-by-trial analysis, WS-related activity was normalized using the response range of each cell (Fi-Fmax/Fmax-Fmin), showing that the WS spike rate correlates inversely with error probability. Normalized activity was plotted against error probability for a corresponding bin size of neuronal activity (bin size = 0.05). Fi, frequency for a given trial; Fmax, maximum rate; Fmin, minimum rate.

The measure of behavioral adaptation was well transcribed by the relative modification of RTs and error rates in post-success and post-error trials. The RTs were not significantly different between post-success and post-error trials (respectively 788.23 and 803.34 ms; *t*-test, *P* = 0.27). However, our study has shown that global behavioral performance (measured for all recording sessions) was clearly influenced by previous trials: the error rate after successful responses (28.7%) was 15.5% higher than post-error behavioral responses (24.2%; χ^2^ test, *P*<0.0001) (Figure 5A). Furthermore, taken individually for each of the recording sessions, this effect was found in a majority of neurons exhibiting a post-error firing rate enhancement in response to the warning stimulus (Table 1; n = 49; 62%; χ^2^ test, *P*<0.001). This is in accordance with a trial-by-trial analysis showing that WS spike rates inversely correlate with error probability (Figure 5B). These results suggest that that enhanced attentional activity positively influences the accuracy of a subsequent behavioral choice; thus the likelihood of making a further erroneous response is reduced by having just committed an error.

**Table 1:** Post-error and post-success effects on both WS-related neuronal activity and behavioral performance.

		WS-Firing rate	
		Post-E>Post-S	Post-E<Post-S
**Error rate**	Post-E<Post-S	49 (62%)	04 (05%)
	Post-E>Post-S	18 (23%)	08 (10%)

Shown are the number and percentage (in parentheses) of neurons after successful or erroneous trials with activity changes subdivided as a function of their corresponding variation in behavioral performances.

## Discussion

We found that after an error, the ACC exhibited an enhanced response to a warning stimulus that increased the accuracy of a subsequent behavioral choice. Our results demonstrate for the first time that both error detection and the subsequent adjustment are processed by single neurons in the ACC. Specifically, the enhancement of alertness is a direct consequence of neuronal changes induced by failure detection. In this section, we will first discuss the possible role of attentional systems in the correction of behavioral error, and, based on our neuronal and behavioral results, we will then define the contribution of the ACC neurons in such a process. Finally, we will propose the possible involvement of ACC dysfunction in the pathophysiology of mental disorders.

Cognitive control must engage performance evaluation but also, and to a less-understood extent, introduce the subsequent implementation of behavioral adjustment mechanisms [15]. Adjustment mechanisms in the SLT engage cognitive control methods such as conflict resolution and are likely to be related to “top-down” control systems such as selective (or executive) attentional processes [16]. Based on the wide distribution of its projections on cortical and subcortical structures, it has been suggested that the ACC plays a direct role in executive attention [17], [18]. While the implications are still under debate [19], [20], neuroimaging studies have partially confirmed this hypothesis [21], [22]. However, the WS responses exhibited by ACC neurons are more related to a phasic alerting attentional system engaged in specific optimization of bodily readiness prior to engaging in further behavior [12], [23], [24]. While the relationship between alertness and executive attentional networks remains unclear [12], [25], this phasic WS-related activity likely influences brainstem noradrenergic neurons [26], which consequently could influence cortical areas involved in selective attentional processes [16]. These results highlight a putative role for the ACC in implementing phasic control over the dorso-lateral prefrontal cortex, which is supposed to represent and actively maintain the attentional demand of a given task [27], [28]. Based on an evolutionary perspective, the assertion that the monkey's ACC has a role in alerting is well in accordance with the view that alertness might be a foundational form of attention on which other attentional systems rest [12].

At the behavioral level, our study has shown that global behavioral performance is improved after erroneous trials, allowing us to propose a relationship between WS processing and behavioral performance adjustment. This confirms the idea that the WS processing enhancement is not always necessary to perform a correct response but rather subserves a behavioral adjustment after a critical situation such as a decrease in vigilance throughout a task or an execution of a faulty response. One other possible strategy for behavioral adjustment has been described in previous experiments on humans by Rabbitt et al. [29], who reported a prolongation of RT after an erroneous response that seemed to be more evident in reaction time tasks. However, we did not find a diminished RT within post-error trials. Because other studies have indicated RT shortening in tasks where a warning stimulus preceded the target [16], we propose that any post-error slowing might have counterbalanced an increased readiness associated with WS processing. This is consistent with the finding that the alerting RT was not affected even when changes in associated neuronal activity were observed [12]. It is also important to realize that numerous factors influence the SLT RT, and previous human studies using a WS have focused on simple reaction time tasks.

On the basis of the bimodal activity patterns of ACC neurons that modulate their discharge rate in response to performance feedback and warning stimulus presentation, our results suggest that both evaluative and attentional ACC activities are intimately linked to error processing. As a consequence, failure detection is immediately followed by an enhancement of attentional resources (i.e., readiness to respond to the upcoming task) that may in turn serve more specific executive control subsystems. This new finding of a neuronal adjustment mechanism is consistent with other studies [30], [31], [32], [33], [34], [35] utilizing different experimental paradigms, suggesting that the ACC provides an error compensation system that serves to modify a subject's behavioral response following an unfavorable and unexpected outcome. This mechanism for remedial action would complement the proposed role of midbrain dopaminergic neurons in preventing the repetition of erroneous responses through a reduction of synaptic weights associated with incorrect behavior [36]. Finally, it is likely that a failure of remedial reaction to error processing as described in this study might be responsible for excessive goal-directed behavior and error perseveration. This may therefore implicate the ACC in obsessive-compulsive disorders (OCD), amongst other psychiatric pathologies [37], [38]. Further studies are needed to explore the modulatory role of afferent catecholaminergic inputs on ACC neurons involved in the processing of arousing stimuli (noradrenaline) [39] and on the prevention of erroneous responses (dopamine) [36]. This question is particularly relevant in order to clarify the biological basis of psychiatric diseases, such as OCD, in which a dysfunction in error processing (obsession) leads to the maintenance of inappropriate behavior (compulsion) [40].

## Materials and Methods

### Ethics statement

Animal care was supervised by veterinarians skilled in the maintenance of non-human primates, in strict accordance with the European Community Council Directive for experimental procedures in animals. All animal procedures conformed to the animal welfare guidelines at University Victor Segalen Bordeaux2 and CNRS institutions and were approved by the local ethics committee 〈〈comite d'ethique sur l'experimentation animale pour la region Aquitaine et Poitou-Charentes〉〉.

### Experimental paradigm

Experiments were performed on two female monkeys (*Macaca mulatta*) weighing 5.5 kg and housed in individual primate cages with free access to food and water. The animals were restrained from drinking the day before experimentation. Monkeys were trained to perform a monkey Stroop-like task (SLT) consisting of touching a color target in response to a visual instruction cue that was simultaneously provided on a video touch-screen. The monkeys learned to associate the shape of a fruit (banana, apple, pear) and a color (yellow, red and green respectively; 5×9 cm). By manipulating the color of the shapes, three conditions were defined: 1) a control situation in which the shape was colorless, 2) a facilitation condition in which the shape had the “correct” color (e.g. the banana was yellow), 3) an interference condition in which there was no correspondence between color and shape (e.g. the banana was red; see Figure 1A).

The 12 different possible combinations were presented randomly within each recording session and the color order of targets (six possibilities; e.g. from left to right: red, green, yellow) was changed for each recording session. The task began with a rest period (3000 ms), during which the monkey's right hand rested on a position sensor, prior to the appearance of a warning stimulus (black circle, diameter 5 cm, 500 ms) that signaled a new trial. After a variable delay (500–1000 ms), the SLT appeared and the monkey had 4000 ms in which to touch the correct target with the same hand in order to get the reward (1 ml orange juice). If the monkey moved prematurely, touched an incorrect target, or failed to respond within the time limit, a black screen was presented for 2000 ms. Both reward delivery and black screen appearance occurred randomly between 500 to 1000 ms after the end of the movement (i.e., after the monkey had replaced its hand on the position sensor). To verify that neuronal responses to the warning stimulus and negative feedback signal were not due to simple visual responses, we presented different visual stimuli such as a colored screen (pink, blue, or brown) or geometrical shapes (square, cross, or triangle). Similarly, drops of orange juice were given out of the context of the task in order to verify that neuronal activity related to reward obtention was not due to simple appetitive mechanisms. The monkeys began the training period (ca. 6 months for each monkey) by learning a simple motor task (further used during subsequent recording sessions as a motor control test), in which they had to touch a black target displayed randomly in left, middle or right screen positions. The control situation was then presented. When the monkey's overall performance reached >50% correct (chance = 33%), the facilitation condition was initiated, followed by the interference condition once the behavioral criterion had been reached.

### Electrophysiology

After completion of the training period, a stainless steel recording chamber (diameter 19 mm) was fixed onto the skull under general anesthesia (ketamine 10 mg/kg, xylazine 2 mg/kg, diazepam 0.5 mg/kg, atropine sulfate 0.2 mg/kg). The center of the cylinder was stereotactically positioned at A26 and L0 on both monkeys in accordance with previously determined MRI localization. A head holder was fixed with dental cement around the chamber in order to immobilize the head of the monkey during neuronal recordings from the left hemisphere. Antibiotic (ampicillin, 100 mg/kg) and analgesic (paracetamol, 30 mg/kg) treatments were given for one week after surgery.

Extracellular single-unit activity was recorded with tungsten microelectrodes insulated with epoxy (impedance 0.5–1.0 MΩ at 1 kHz). Neuronal activity was amplified (10–20 K), filtered (300 Hz–3 KHz) and displayed on an oscilloscope. Spikes were selected from background activity with a window discriminator before being processed though an analogue-digital interface and stored on-line on a microcomputer. The activity of single neurons was compared with respect to various events and outcomes resulting from different conditions. This was achieved by convolving spike trains with a combination of growth and decay exponential functions that resembled a postsynaptic potential [7], [41].

### Behavioral analyses

Measurements were made of the behavioral reaction time (RT), which was defined as the interval between the SLT screen appearance and release of the position sensor. The error rate was calculated for each trial condition by dividing the number of errors by the total number of trials (i.e. errors+successes). Only those trials for which touching movements were completely achieved were included in the analyses.

### Histology

At the end of experimentation, histological preparations were made using techniques previously described [42]. Reconstruction of the recording sites was based on the coordinates of each recorded cell with respect to marker lesions and, when possible, to the electrode tracks (see Figure 1B). CMAr boundaries were determined with sulcal landmarks as well as information obtained by intracortical microstimulations performed after each neuronal recording.

### Statistical analyses

Statistical analyses were performed during two different epochs. The beginning and end of each epoch were defined by the onset and offset of the respective stimulus. In this way, the length of a given epoch corresponded to the total stimulus duration (i.e. warning stimulus, 500 ms; reward delivery, 1000 ms and negative signal feedback, 2000 ms). Neurons whose firing rate during a given epoch was significantly different (paired *t*-test, P<0,01) from their baseline firing rate during the rest period were defined as expressing task-related changes in activity. Unpaired t-tests were then performed to differentiate the different performance conditions (success, error, post-success, post-error) for each task-related neuron. The post-error condition accounted only for errors performed in trials for which touching movements were completely achieved. The neuronal discharge rate (spikes/s) was obtained by calculating the average firing rate during a 200 ms window centered 100 ms around the highest activity of the spike density function histograms. The maximum peak firing of task-related neurons was then measured within each of these reduced 200 ms epochs, averaged, and compared with a paired t-test in order to determine whether CMAr neurons, as a population, could differentiate between the different conditions. Since no statistical differences were found between data collected from each monkey, results were pooled for further analysis.
